# Report of the Commissioners of Agriculture

**Published:** 1883-04

**Authors:** 


Report of the Commissioner of Agriculture for the years 1881 and 1882. 8vo., 704 pages—plates. Washington, 1882.

For the first time in a number of years this report is issued sufficiently early to be of service to its readers. The portion devoted to veterinary subjects is particularly interesting at the present time, containing as it does Dr. D. E. Salmon's report on the swine plague, fowl-cholera, and Southern cattle fever. In regard to fowl-cholera the Doctor decides that the disease is introduced in some tangible form into the bodies of the healthy fowls by way of the digestive organs.

The other articles are a report on the Swine Plague and Diseases among Horses, by Dr. H. J. Ditmers; Contagious Pleuro-Pneumonia, by Charles P. Lyman, F.R.C.V.S.; and Anthrax among Cattle in New Jersey, by Dr. Ezra M. Hunt.

